# C/EBP Transcription Factors in Human Squamous Cell Carcinoma: Selective Changes in Expression of Isoforms Correlate with the Neoplastic State

**DOI:** 10.1371/journal.pone.0112073

**Published:** 2014-11-17

**Authors:** Sanjay Anand, John Ebner, Christine B. Warren, Manu S. Raam, Melissa Piliang, Steven D. Billings, Edward V. Maytin

**Affiliations:** 1 Department of Dermatology, Cleveland Clinic, Cleveland, Ohio, United States of America; 2 Department of Biomedical Engineering, Cleveland Clinic, Cleveland, Ohio, United States of America; 3 Department of Anatomic Pathology, Cleveland Clinic, Cleveland, Ohio, United States of America; 4 Wellman Center for Photomedicine, Massachusetts General Hospital, Harvard Medical School, Boston, Massachusetts, United States of America; Università degli Studi di Milano, Italy

## Abstract

The CCAAT/Enhancer Binding Proteins (C/EBPs) are a family of leucine-zipper transcription factors that regulate physiological processes such as energy metabolism, inflammation, cell cycle, and the development and differentiation of several tissues including skin. Recently, a role for C/EBPs in tumor cell proliferation and differentiation has been proposed, but the incomplete characterization in the literature of multiple translational isoforms of these proteins has made interpretation of these roles difficult. Therefore, we have carefully reexamined C/EBP isoform expression in human non-melanoma skin cancers. C/EBPα, C/EBPβ, and C/EBPδ were analyzed histologically in squamous cell carcinomas (SCC). The individual isoforms of C/EBPα and C/EBPβ were examined by immunofluorescent digital imaging, western blotting and DNA binding activity (electrophoretic mobility shift analysis). Expression of all C/EBP family proteins was decreased in SCC tumors. Suppression was greatest for C/EBPα, less for C/EBPβ, and least for C/EBPδ. Western analyses confirmed that C/EBPα p42 and p30 isoforms were decreased. For C/EBPβ, only the abundant full-length isoform (C/EBPβ−1, LAP*, 55 kD) was reduced, whereas the smaller isoforms, C/EBPβ−2 (LAP, 48 kD) and C/EBPβ−3 (LIP, 20 kD), which are predominantly nuclear, were significantly increased in well- and moderately-differentiated SCC (up to 14-fold for C/EBPβ−3). These elevations correlated with increases in PCNA, a marker of proliferation. Although C/EBPβ displayed increased post-translational modifications in SCC, phosphorylation of C/EBPβ−1 (Thr 235) was not altered. C/EBP-specific DNA binding activity in nuclear and whole-cell extracts of cultured cells and tumors was predominantly attributable to C/EBPβ. In summary, two short C/EBPβ isoforms, C/EBPβ−2 and C/EBPβ−3, represent strong candidate markers for epithelial skin malignancy, due to their preferential expression in carcinoma *versus* normal skin, and their strong correlation with tumor proliferation.

## Introduction

C/EBP transcription factors (C/EBPs), a family of six gene members in which C/EBPα, C/EBPβ, and C/EBPδ have been the most intensively studied, were originally identified as regulators of growth and differentiation in normal tissues [1]–[3]. More recently, C/EBPs have received considerable attention as potential molecular markers that define prognostic risk in cancer; reviewed in [4]–[6]. C/EBPα is known to be a tumor suppressor in acute myelogenous leukemia [reviewed in [7], [8]], and is reportedly down-regulated in human epithelial cancers of the breast [9], lung [10], liver [11], head and neck [12], endometrium [13], and squamous cell carcinoma (SCC) of the skin [14]. C/EBPα expression levels in actinic keratoses and keratoacanthoma (precancerous skin conditions that can progress to SCC) were reduced relative to normal epidermis, and fully invasive SCCs expressed no detectable levels of C/EBPα, indicating a direct correlation between expression levels of C/EBPα and the severity of neoplasia [14]. C/EBPβ expression, in contrast to C/EBPα, was reportedly increased in advanced cases of breast, ovarian, colorectal, renal, and gastric carcinoma [15]–[21]. However, interpretation of those reports is complicated by the existence of three different isoforms of C/EBPβ, a major topic of the current study (*see below*). C/EBPδ gene has been reported to be methylated in acute myelomoncytic leukemia (AML), cervical, breast and hepatocellular carcinoma and reduced expression associated with progression of breast tumors [22]. Other C/EBP family genes have received relatively less attention, but potential involvement of dysregulated C/EBPε [23], and C/EBPζ/gadd153 [24] in leukemia and melanoma, respectively, has been suggested.

The question of whether absolute protein levels of the various C/EBPs reflect biological functions in human cancers remains unresolved, but it appears increasingly clear that changes in the ratio of C/EBP isoforms could have prognostic significance. C/EBPα and C/EBPβ are intronless genes, each producing a single mRNA transcript. Different protein isoforms can be produced from a single C/EBPα or C/EBβ transcript via a mechanism of alternative translation that uses different ATG start codons within the ribonucleotide sequence [25]. The human C/EBPα transcript produces two proteins, ∼42 kDa and ∼30 kDa, whereas the C/EBPβ transcript produces three proteins named LAP*, LAP, and LIP in mice, or C/EBPβ-1, C/EBPβ-2, and C/EBPβ-3 in humans, in order of decreasing size [6]. The shortest form can act as a dominant-negative inhibitor since it contains a DNA-binding region, yet lacks N-terminal amino acids necessary to transactivate gene transcription. For this reason, the relative expression levels of a long and short form, the “LAP/LIP ratio,” was proposed to participate in regulation of proliferation and differentiation in normal cells [26], [27], and in cancer [28]. This idea now deserves a revisit as more detailed information has emerged. Linda Sealy et al. have established that the largest form of C/EBPβ observed in transformed breast cancer cell lines is C/EBPβ-2 (LAP), whereas the full-length C/EBPβ-1 (LAP*) is only expressed in whole tissues *in vivo* or in primary epithelial cells *in vitro* [29], [30]. Also, elevated expression of C/EBPβ-2 in MCF10A normal human mammary epithelial cells results in transformation, an epithelial to mesenchymal transition (EMT) and acquisition of an invasive phenotype, directly linking C/EBPβ-2 to severity of neoplasia [31]. The implications here are two-fold: (i), current information about C/EBPβ-1 is not clear, given many older reports in which the analyses were performed immunohistologically and distinction between C/EBPβ-1 and C/EBPβ-2 was not recognized; (ii), previous interpretations regarding proteins extracted from tissues *in vivo* and detected by western blotting may need to be reconsidered, in light of newer information.

Murine models of cutaneous carcinogenesis may offer particular insight because recent reports have indicated important functional roles for C/EBPα and C/EBPβ during tumorigenesis in murine skin. Mice lacking C/EBPα in the epidermis show normal proliferation and differentiation but were highly susceptible to skin tumorigenesis in response to carcinogens. These mice displayed decreased tumor latency, increased tumor incidence, multiplicity, growth rate and malignant progression [14], [32]. Chemically induced squamous cell carcinomas and also primary cell lines established from these SCC, showed negligible expression of C/EBPα as compared with normal epidermis [33]. Also, the expression levels of C/EBPα or C/EBPβ were a direct indicator of the state of neoplasia, since benign papillomas in mice showed an intermediate expression level, when compared to normal epidermis (highest level) and SCCs (lowest level) in the same study [34]. C/EBPβ knockout mice are completely refractory to skin tumor development in response to chemical carcinogens [35] or UVB exposure [Anand *et al*., unpublished results]. A robust increase in apoptosis accompanied with elevated p53 levels in epidermis has been suggested as the causative mechanism for this resistance to tumor formation in C/EBPβ null mice [36]. In a similar study, C/EBPδ KO mice showed no difference in the tumor phenotype as compared to wild type, in response to chemical carcinogen [37]. However, in murine model of mammary tumerigenesis, loss of C/EBPδ resulted in increased mammary tumor multiplicity and reduced lung metastasis involving the regulation of HIF-1α, mTOR and FBXW7 [22], [38]. The role of C/EBPδ as an inflammatory response gene and also a candidate tumor suppressor gene has been supported by the sensitivity of C/EBPδ null mice to ionizing radiation-induced hematopoietic and intestinal injury [39].

In this paper, we have re-examined the question of change in histological expression of the three most abundant C/EBPs (α, β and δ) in human SCC, by looking comprehensively *in vivo* at expression of individual protein isoforms and their DNA-binding ability. Our data confirm an across-the-board downregulation of C/EBPα but more interestingly, the data show a strong upregulation of C/EBPβ-2 and C/EBPβ-3 that correlates with cellular proliferation in moderately- and well-differentiated SCC of the skin.

## Materials and Methods

### Culture of primary keratinocytes and carcinoma cells

Normal human epidermal keratinocytes (NHEKs; Cascade Biologics, Portland, OR); HEK1 cells (HEK001; from ATCC, Manassas, VA); and SCC13 cells (gift from Jonathan Garlick, Tufts University, Boston, MA, [40], [41]) were cultured at 37°C in a humidified CO_2_ incubator as previously described [42]. Human prostate carcinoma cells (LNCAP; ATCC) were cultured as described [43]. *cos-7* cells (ATCC) were cultured at 37°C in a 5% humidified CO_2_ incubator in DMEM (4 mM L-glutamine, 1.5 g/l sodium bicarbonate, 4.5 g/l glucose, with10% FBS, 100 units/ml Penicillin and 100 µg/ml streptomycin). Cells were maintained at subconfluence (<80–90%) levels, and seeded at a 1∶6 to 1∶8 dilutions during passaging.

### Collection of human tumor specimens

Discarded skin tissues, taken during Moh’s surgery for removal of SCC tumors diagnosed previously by biopsy, were obtained in a de-identified manner. This study was approved by the Institutional Review Board of the Cleveland Clinic. Frozen tissue blocks in OCT compound (Tissue Tek, Torrance, CA) were screened histologically, and those that contained usable tumor were stored at −80°C until further study.

### Expression vectors, cell transfection and preparation of whole-cell and nuclear extracts

Expression vectors (pcDNA3.1) encoding rat C/EBPα and mouse C/EBPδ were kind gifts of David Ron (NYU Medical Ctr, New York, NY) and James Dewille (Ohio State University, Columbus, OH), respectively. An expression vector (pCMV6) encoding human C/EBPβ was purchased from Origene (Rockville, MD). An expression vector for human C/EBPβ1 (pcDNA3.1hisA) that exclusively expressed the longest form of C/EBPβ due to modification of the ATG for C/EBPβ2 was kindly provided by Linda Sealy (Vanderbilt University, Nashville, TN). Nuclear extracts from *cos-7* cells overexpressing various C/EBPs (α, β and δ) were prepared exactly as described by Schreiber et al. [44] following transfection of cells in 100 mm dishes using 3 µg of expression vector and GenePORTER reagent (Genlantis, San Diego, CA) according to the manufacturer’s protocol. Whole cell extracts from cells overexpressing the C/EBPs were prepared by lysis in urea buffer (7 M urea, 2% IGEPAL, 5% β-mercaptoethanol and protease inhibitor cocktail), followed by 3–5 pulses (4–6 sec each) of sonication to disrupt membranes, and a 5 min high-speed microfuge spin to clear the lysates. All procedures were carried out at 4°C [45].

### Histological and immunofluorescent analysis of C/EBP expression in human tumors

Cryosections (5 µm) of frozen tumor samples were fixed 5 min in ice-cold methanol and stained with hematoxylin and eosin by standard methods. For immunohistochemical detection of C/EBPs, methanol-fixed cryosections were washed in PBS, and permeabilized in 0.1% Triton X-100 (Sigma, St Louis, MO) in PBS for 10 min on ice. Sections were serially incubated in the following solutions: 3% normal donkey serum, 30 min, room temperature (RT); primary antisera, overnight, 4°C; PBS rinses, 5 min x 3; Cy3-conjugated donkey anti-rabbit IgG (Jackson ImmunoResearch, West Grove, PA, 1∶1500, for 4 h at RT), PBS rinse x1; mounting in Vectashield (Vector Lab, Burlingame, CA) under coverslips. Antisera for C/EBPα, C/EBPβ, C/EBPδ, E-cad (all from Santa Cruz Biotechnology, Santa Cruz, CA) and Ki67 (Thermo Fisher, Waltham, MA) were used at 1∶50 dilution in PBS.

### Semiquantitative analysis of fluorescence intensity in immunostained sections of human skin tumors

Imaging of fluorescence intensity from immunostained skin specimens was standardized as follows. Seven different exposures of a given tumor specimen, and of a normal skin specimen from the same patient, was digitally captured on an Olympus BX50 fluorescent microscope (Olympus America, Center Valley, PA) equipped with a OolSNAP-Pro color CCD camera (Media Cybernetics, Bethesda, MD). Exposure times for each successive image were lengthened so as to double the amount of light collected. Pairs of digital images from tumor and normal hair follicle were compared side-by-side on a computer monitor, and the two images whose intensities were visually most closely matched were noted. From the ratio of exposure times of the best-matched image pair, the relative difference (fold) in C/EBP protein between tumor and normal was determined. For this analysis it was assumed that the fluorescent signal is directly proportional to the amount of C/EBP protein bound by primary antibody, since no amplification step was employed during immunostaining. Some examples of images analyzed in this way are illustrated in **Fig. S2**.

### Western blot analyses

Cells were lysed and for human tumor samples, 30 µm cryosections (cut perpendicular to the skin surface) were scraped from the glass slide and pooled, then homogenized, lysed and sonicated in urea lysis buffer as described [42]. Protein content was determined by Bradford assay kit (Bio-Rad, Hercules, CA), and equal amounts were analyzed on western blots using 4–12% Bis-Tris acrylamide mini gels (Invitrogen, Carlsbad, CA) or 10% Tris-Glycine gels, as described [42]. The source and dilution of antisera used here were as follows: C/EBPα, C/EBPβ, C/EBPδ, Actin and GAPDH (Santa Cruz,1∶5000); PCNA and E-Cadherin (Santa Cruz, 1∶2000); α-Tubulin (Sigma,1∶10000), Phospho-C/EBPβ (Cell Signaling, Danvers, MA, 1∶1000); and peroxidase-conjugated goat anti-rabbit IgG (Jackson ImmunoResearch, 1∶20,000). Western blot signals were quantitated using IPLab software (Scanalytics Inc., Fairfax, VA).

### Electrophoretic Mobility Shift Assay (EMSA)

Complementary oligonucleotides (oligos; 29-mer) that spanned either a well-established C/EBP motif, or a mutant C/EBP motif, were synthesized (Integrated DNA Technologies, San Diego, CA) and used for EMSA. The sense and antisense sequences for each of the oligos are as follows:

C/EBP consensus; (Sense 5′ CTAGCATCTGCAG**ATTGCGCAAT**CTGCAC 3′; Antisense 5′ TCGAGTGCAG**ATTGCGCAAT**CTGCAGATG 3′).

Mutant C/EBP consensus; (Sense 5′ CTAGCATCTGCAG**AGGTATACCT**CTGCAC 3′; Antisense 5′ TCGAGTGCAG**AGGTATACCT**CTGCAGATG 3′). The C/EBP consensus sequence is shown in bold, and mutant sequences are underlined [46]. Two pM of duplex oligos, heat denatured and annealed, were labeled with [α-^32^P] dCTP (3,000 Ci/mM, ICN Pharmaceuticals) using Klenow polymerase (New England Biolabs, Ipswich, MA) and purified using Probequant spin columns (GE Healthcare, Piscataway, NJ). Nuclear extracts (2–3 µg) from *cos-7* cells, keratinocytes, or tumor cells were prepared as described [44] and incubated with 50 fM of labeled oligos in DNA binding buffer (Hepes pH 7.9, 20 mM, glycerol 10%, KCl 40 mM, NP-40 0.1%, EDTA 0.5 mM, PMSF 1 mM, DTT 0.5 mM) along with 1 µg of non-specific DNA competitor [poly(dG-dC)**^.^** poly (dG-dC)] and 1 µl of filtered FBS for 30 min at room temperature. DNA-protein complexes were resolved on 4% non-denaturing polyacrylamide gels in Tris-Glycine-EDTA buffer (Tris 25 mM, Glycine 200 mM and EDTA 2.25 mM), dried and exposed to X-ray film between intensifying screens. For supershift experiments, 1 µl of antibody was added to the mixture and incubated for 15 min prior to addition of labeled oligos [45].

### Statistical analyses

**Figure 1:**
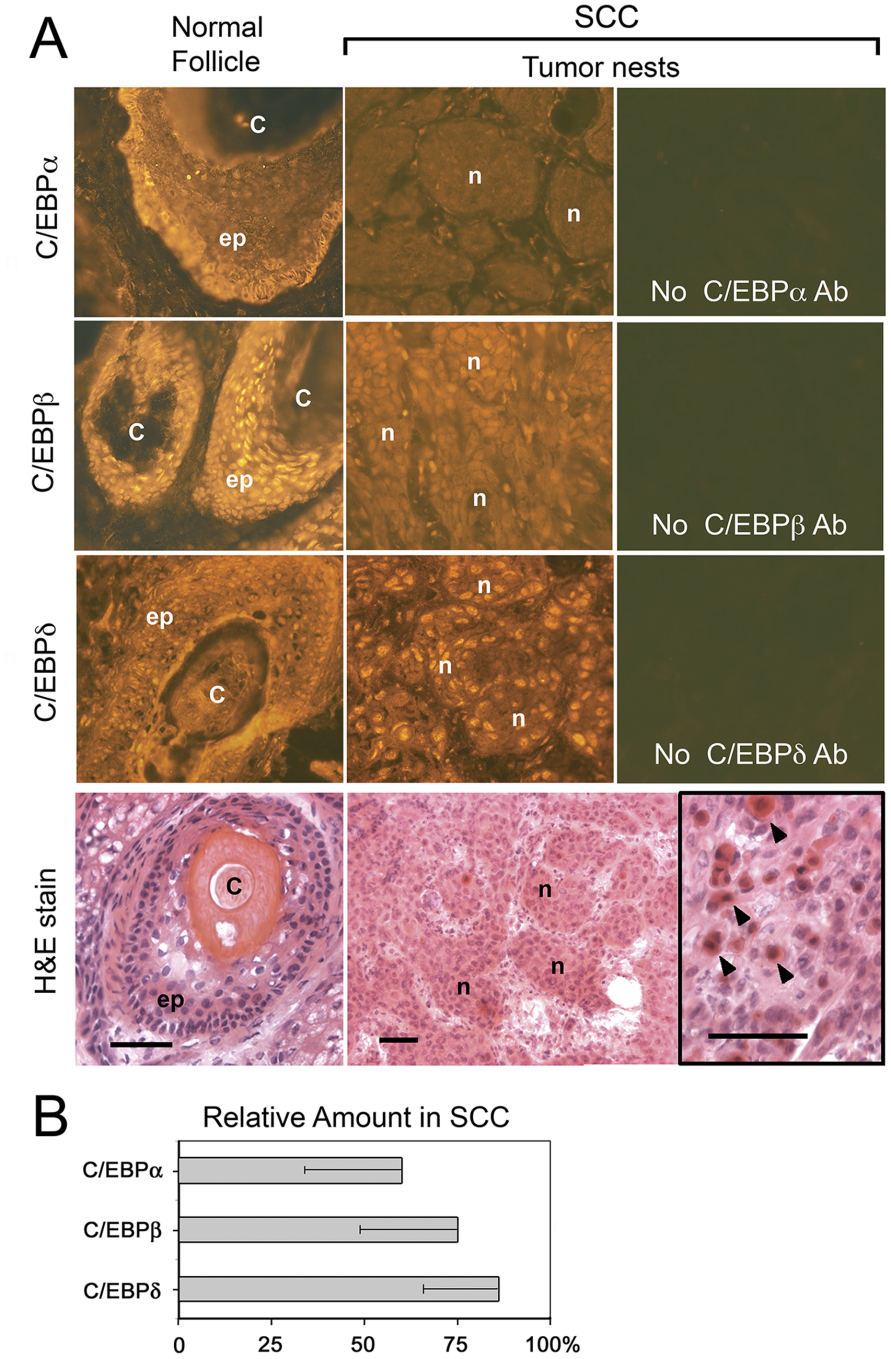
Expression patterns of C/EBPα, C/EBPβ, and C/EBPδ in human squamous cell carcinoma (SCC). (**A**) Frozen skin sections from patients with SCC were fluorescently immunostained, using antibodies to C/EBPα, C/EBPβ, or C/EBPδ (as indicated on left) followed by a Cy3-fluorotagged anti-IgG secondary antibody. Hematoxylin and eosin (H&E) stains were done on parallel sections. Tumor nests (*n*), and normal hair follicle epithelium *(ep)* and cortex *(C)*, are indicated. *Inset: Arrowheads* indicate well-differentiated squamous cells, expressing high levels of keratins (*red*). *Scale bars*, 50 µm. (**B**) Quantification of fluorescent staining intensities for the three C/EBP family proteins (α,β and δ) in eleven SCC tumors, relative to normal follicles; mean ± SD.

**Figure 2:**
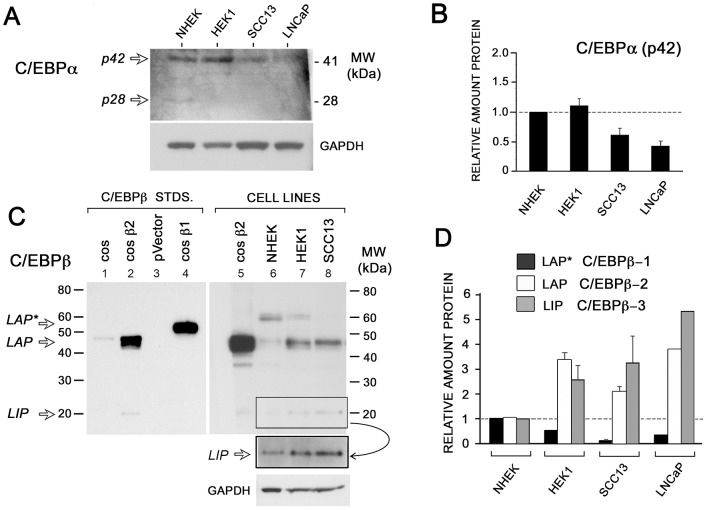
C/EBPα and C/EBPβ isoforms are differentially expressed in SCC cell lines as compared to normal human keratinocytes. Western analyses of normal primary keratinocytes (NHEK), and two squamous cell carcinoma cell lines, HEK1 and SCC13. (**A**) Western blot for C/EBPα, in the skin cell lines and also in prostate carcinoma cells (LNCAP). GAPDH is a loading control. (**B**) Densitometric quantification for C/EBPα. (**C**) Western blots for C/EBPβ. Lanes 2 and 5 (cos β2) contain recombinant human cDNA for C/EBPβ overexpressed in cos-7 cells, which translate only LAP and LIP. Lane 4 (cos β1) contains recombinant human T7 his-tagged C/EBPβ-1 plasmid expressed in cos-7 cells. Lanes 1 and 3, extracts from cos-7 cell that were untransfected or transfected with the empty vector (pVector), respectively. *Box*, longer exposure of C/EBPβ-3. GAPDH, loading control. (**D**) Densitometric quantification of each isoform of C/EBPβ. Graphs represent the average of two independent Western analyses, with protein levels normalized to GAPDH and expressed relative to NHEK (*dotted line*).

Relative levels of C/EBPs (compared to normal skin) were quantitated using immunohistological images (three images per sample) from 10–13 SCC samples and data presented as mean±SD in **Table 1** and **Fig. 1B**. Protein expression levels by western blot were quantitated using blots from two independent experiments and the data presented as average±range in **Figs. 2** **–** **4**.

**Figure 3:**
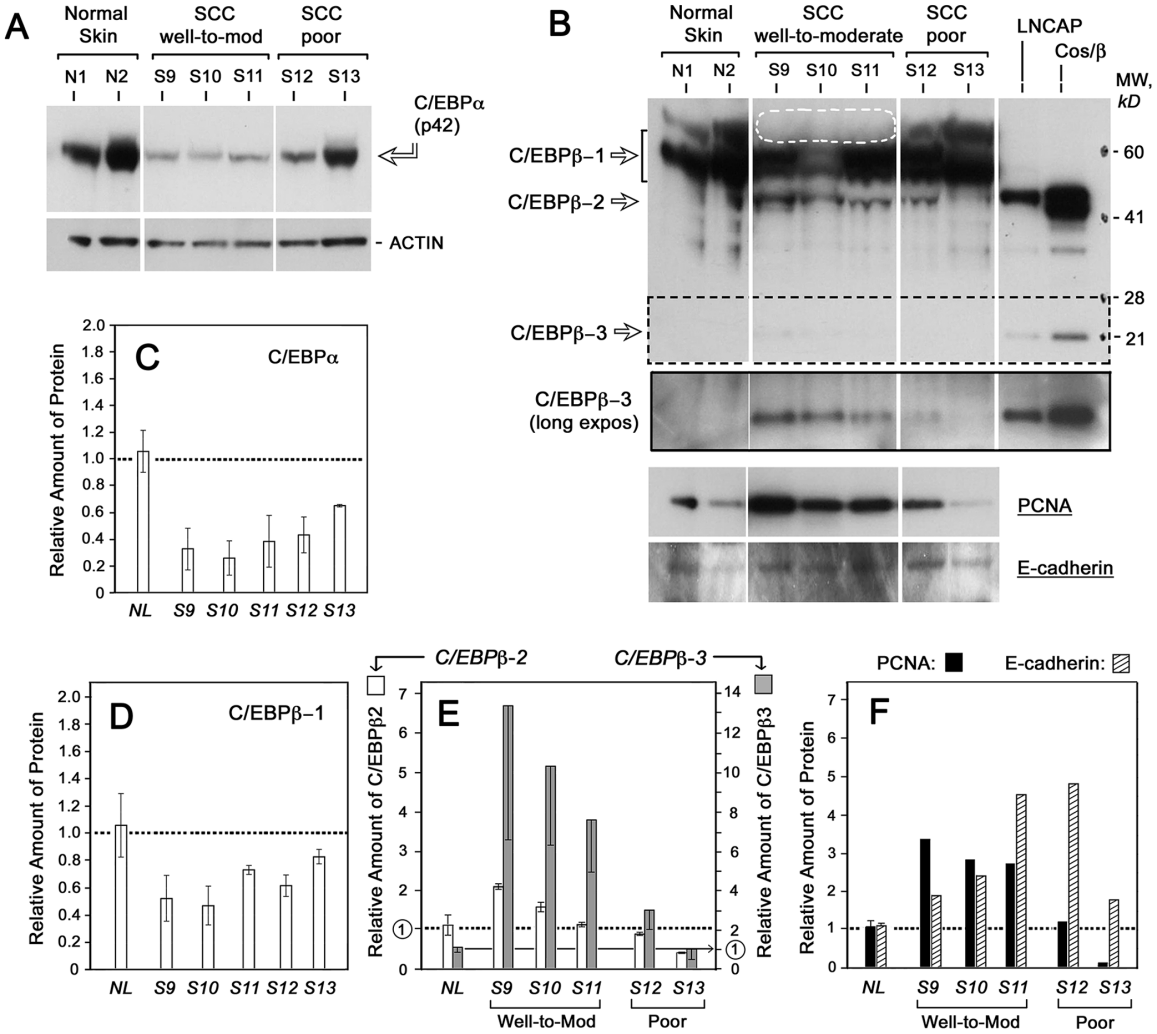
C/EBPα and C/EBPβ expression in human skin SCC. Total protein lysates from normal skin samples (N1, N2), three moderately- or well-differentiated squamous carcinomas (S9, S10 and S11), and two poorly-differentiated squamous cell carcinomas (S12 and S13) were analyzed on western blots. Blots for tubulin and actin were done as loading controls for all samples; only actin is shown. (**A**) Western analyses for C/EBPα. (**B**) Western analyses for C/EBPβ. The last two lanes are positive controls, consisting of lysates from a prostate cancer cell line (LNCAP) and from cos-7 cells transfected with a native full-length sequence of human C/EBPβ (cos/β). *Solid box*, longer exposure of region within *dotted lines*. At bottom, western blots for PCNA and E-cadherin. (**C**) Densitometric quantification of C/EBPα. Duplicate gels were scanned, and the range shown by the error bars. (**D**) Densitometric quantification of C/EBPβ−1 isoform. (**E**) Densitometric quantification of C/EBPβ−2 and C/EBPβ−3 isoforms. Note that for C/EBPβ-3, the y-axis scale is on the right. (**F**) Densitometric quantitation of markers for proliferation (PCNA) and differentiation (E-cadherin). All band intensities were normalized to the average of actin and tubulin (not shown). *Error bars*, range of duplicate measurements.

**Figure 4:**
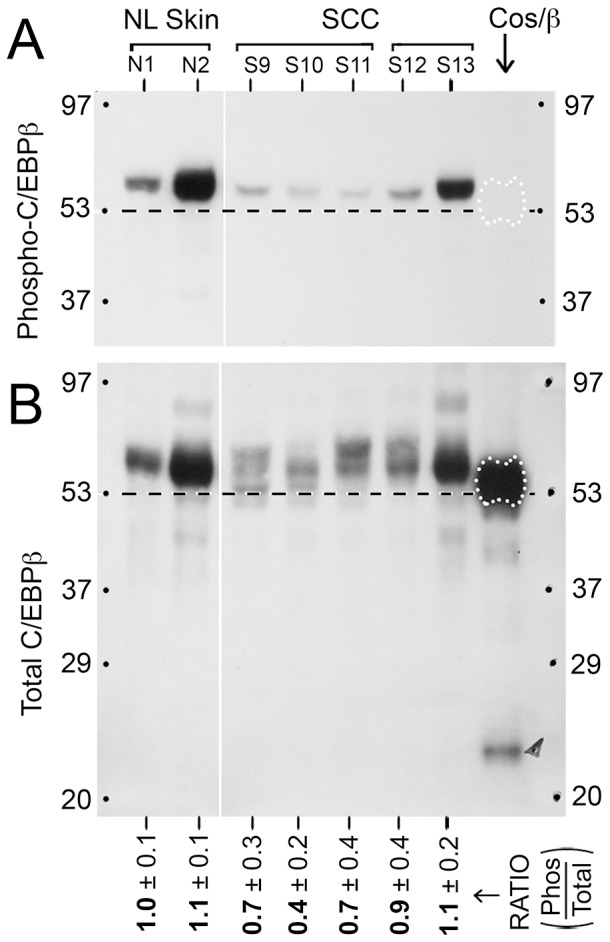
C/EBPβ-1 is phosphorylated at threonine-235 in human SCC. Western blots from the same lysates used in **Fig. 3** were run and developed with antibodies to either: (**A**) phospho-C/EBPβ (Thr235), or (**B**) total C/EBPβ. The last lane contains lysates from cos-7 cells transfected with full-length human C/EBPβ vector. Dashed lines indicate the approximate location of recombinant C/EBPβ-2 to facilitate comparisons between the tumor lysates. MW markers in kD are indicated. *Bottom,* ratio of phosphorylated-to-total C/EBPβ (mean ± range) from densitometry of two western blot analyses.

**Table 1:** Relative levels of C/EBPα, C/EBPβ and C/EBPδ in histological sections of human squamous cell carcinoma.

Protein Expression (% of normal)			
Tumor ID#	C/EBPα	C/EBPβ	C/EBPδ
S1	ND	100	83
S2	53	80	100
S3	ND	43	100
S4	61	100	53
S5	43	55	66
S6	100	66	100
S9	45	100	111
S10	31	100	100
S11	33	42	66
S12	71	100	100
S13	ND	40	64
***Mean ± SD***	**60**±26	**75**±26	**86**±20

The relative intensity of immunofluorescent staining (compared to normal skin) for each C/EBP family member was determined semi-quantitatively by making pairwise visual comparisons between tumor nests and normal hair follicles within the same patient, using the f-stop technique (see Methods and Figure S2). *ND*, not done.

## Results

### Expression of C/EBPα, C/EBPβ, and C/EBPδ is reduced in human cutaneous SCC

To obtain qualitative information about expression levels and intracellular localization of C/EBP transcription factors in skin cancers *in vivo*, we used primary antibodies to C/EBPα, C/EBPβ, and C/EBP**δ** to perform immunostaining on frozen SCC specimens and adjacent normal skin (**Fig. 1**). Staining patterns for C/EBPα and C/EBPβ in normal epidermis, hair follicles, and sebaceous glands were consistent with previous reports [47], [48] i.e. C/EBPα and C/EBPβ staining was primarily cytoplasmic in the lower epidermal layers and hair follicle epithelium (**Fig. 1A**, “ep”), but became nuclear in the upper epidermal layers. The difference between C/EBPα and C/EBPβ was that greater amount of cytoplasmic C/EBPβ was also seen in the normal follicles (**Fig. 1A**). For C/EBPδ, expression was strongly nuclear throughout all layers of the epidermis (not shown) and in the follicles (**Fig. 1A**).

Within SCC tumor nests, the signal intensity for each C/EBP protein was markedly lower in tumor cells than in normal cells (**Fig. 1A**), with some exceptions. To obtain more objective estimates of changes in C/EBP protein expression in the biopsy specimens, we devised an “f-stop” technique to quantify changes in the immunofluorescent signal in digitally-recorded micrographs; see Methods and **Fig. S2**. The graphs in **Fig. 1B** summarize the results of this enhanced analysis for multiple SCC tumors; data for the individual tumors are in **Table 1**. For SCC, C/EBPα was suppressed in 7/8 specimens, C/EBPβ in 6/11 specimens, and C/EBPδ in 5/11 specimens. Thus, in terms of both frequency and magnitude of change in expression, C/EBPα was suppressed the most, C/EBPβ less so, and C/EBPδ the least.

### Both C/EBPα isoforms and CEBPβ-1 isoform are decreased, whereas CEBPβ-2 and CEBPβ-3 are increased in malignant SCC cell lines

Because standard immunohistological technique can not differentiate between isoforms, we used western analysis to further refine our initial observations of C/EBP protein expression in SCC, focusing on C/EBPα and C/EBPβ. For a preliminary look, and to confirm the specificity of antibody reagents, we tested several cell lines that lie along a spectrum of increasingly malignant behavior. In the order of benign to malignant, the lines were: (i), NHEK, a normal human epidermal keratinocytes, (ii), HEK1, a virally transformed human keratinocyte line, (iii), SCC13, a spontaneously-tranformed squamous cell carcinoma line, and (iv), LNCAP, a prostate carcinoma line. In these cell lines, expression profiles of markers of growth-arrest and differentiation had already been established [see Fig. S1 in ref. [42]]. Here, western analyses revealed a decrease in both C/EBPα isoforms (42 kD and 30 kD) in parallel with the hierarchy of malignant progression (**Fig. 2A, B**). This finding was consistent with clinical reports in breast carcinoma [9], human SCC [14] and experimental cutaneous carcinoma in mice [33], [49]. For C/EBPβ, the situation was more complicated. Because previous literature did not always distinguish between the two large isoforms of C/EBPβ, we took care to confirm the identity of C/EBPβ-1 and C/EBPβ-2 on gels by comparing their location on the western blots with the location of recombinant proteins translated in cos-7 cells. For calibration purposes, we used: (i), a normal full-length human C/EBPβ sequence that expresses C/EBPβ-2 and −3 but not C/EBPβ-1 in the cos-7 cells (**Fig. 2C**, lanes 2 and 5), and (ii), a cDNA engineered to express only C/EBPβ-1 and not the other isoforms (lane 4). In the cell lines, the abundant C/EBPβ-1 isoform appeared to decrease with malignant progression (**Fig.** **2C**, lanes 6–8), whereas the two smaller isoforms C/EBPβ-2 and C/EBPβ-3 were increased 2- to 5-fold (**Fig.** **2D**). These results agree with others who reported that C/EBPβ-1 is expressed in normal mammary cells and tissues, but not expressed in immortalized cell lines. These data are also consistent with reports that high C/EBPβ-2 expression can results in transformation, EMT and acquisition of an invasive phenotype in normal human mammary epithelial cells [29], [31]. We also tested for C/EBPδ, but signals on Western blots were too weak to assess. Low expression levels of C/EBPδ in these cell lines were later confirmed by functional DNA binding assays (see below).

### In human SCC cancers, overall expression of C/EBPα and C/EBPβ is reduced, but expression of two small C/EBPβ isoforms is increased

We next examined human SCC specimens by western blotting. Obtaining sufficient tumor tissue from skin biopsies was only possible by pooling multiple thick frozen sections from the largest tumors, which comprised five invasive SCC. Those specimens were evaluated by two certified dermatopathologists to determine the histological tumor grade (**Fig. S1**). Protein lysates from these tumors were loaded onto western blots in the order of worsening neoplasia, using duplicate blots to provide an estimate of variability (**Fig. 3**). Three proteins (GAPDH, tubulin and actin) were used as housekeeping loading controls, instead of only one, because expression of “invariant” control genes often differs between skin samples as demonstrated by Minner and Poumay [50]. **Fig. 3A** illustrates a typical Western blot for C/EBPα in normal and carcinoma tissues (only the actin control is shown). C/EBPα (p42) expression decreased by 60–75% in most of the SCCs, although less so in the most poorly differentiated tumor (S13). The C/EBPα p30 isoform was not detected.

For C/EBPβ, each isoform behaved differently. The largest protein, C/EBPβ-1, appeared as a thick multi-banded complex (55–70 kD), indicating a high level of post-translational modification (**Fig. 3B**), as described by Zahnow [6]. The abundance of C/EBPβ-1 was moderately reduced in all SCC (**Fig. 3D**), although a more remarkable loss of the highest MW (most post-translationally modified) forms of C/EBPβ-1 was absent in the well-to-moderately differentiated SCC tumors, S9–S11 **(** **Fig. 3B**, dotted oval). In contrast, the shorter isoforms, C/EBPβ-2 and β-3, were both strongly induced in tumors S9–S11 (**Fig. 3B**). Although expressed in low amounts, C/EBPβ-2 and C/EBPβ-3 differed qualitatively from C/EBPβ-1 in that both were undetectable in normal skin. By densitometry, C/EBPβ-2 and C/EBPβ-3 were increased in the well- and moderately-differentiated SCC, but not in the poorly-differentiated SCC (**Fig. 3E**). C/EBPβ-3 appeared to offer the greatest signal-to-noise ratio, rising 8- to14-fold in the moderately-differentiated tumors (**Fig. 3E**).

### The expression patterns of C/EBPβ-2 and C/EBPβ-3 in cutaneous SCC correlate with changes in markers of cellular proliferation and differentiation

To ask whether changes in the short C/EBP isoforms were correlated with any physiologic markers within tumors, markers of proliferation (PCNA) and differentiation (E-cadherin) were analyzed in the SCC by western blot (**Fig. 3B**, bottom two panels). PCNA expression levels tracked very closely with expression of the smallest isoform, C/EBPβ-3, such that PCNA and C/EBPβ-3 were elevated in the same tumor subset (**Fig. 3E and 3F**). E-cadherin did not bear any clear relationship with C/EBPβ expression. However, within SCC tumors as a group, E-cadherin expression rose as PCNA expression dropped (**Fig. 3F**), consistent with a permissive relationship between growth arrest and the onset of terminal differentiation often seen in squamous epithelia. Immunohistological analyses of proliferation (Ki67) and differentiation (E-cad) in SCC revealed a similar pattern as described above (**Fig. S3**).

### The C/EBPβ-1 isoform is phosphorylated in SCC, but phosphorylation does not appear to be differentially regulated

To begin to examine post-translational modifications of C/EBPβ, we evaluated the phosphorylation state of C/EBPβ by probing blots with a selective antibody against C/EBPβ phosphorylated at threonine-235 (**Fig. 4A**). Phosphorylation of the threonine 235 residue in human C/EBPβ by Ras-MAPK-ERK kinase (MEK) signaling pathway results in transcriptional activation of C/EBPβ [6]. Here only one band corresponding to phosphorylated C/EBPβ-1/LAP*, was detected. However, up to four bands corresponding to various other forms of C/EBPβ-1 were apparent in the tumor specimens (**Fig. 4B**). In normal skin, only one major form of C/EBPβ-1 was seen (**Fig. 4B**, lanes 1 and 2) and it corresponded to phosphorylated C/EBPβ-1. Slower-migrating forms of C/EBPβ were observed in the SCC tumors and may correspond to acetylation, methylation, or sumoylation, as reported by others and summarized by Zahnow [6]. The ratio of phosphorylated C/EBPβ-1 to total C/EBPβ-1 (defined as those isoforms located above the dotted line in **Fig. 4B**) did not change significantly in SCC relative to normal skin.

### DNA-binding studies in SCC cell lines and tumor biopsies suggest a functional predominance of C/EBPβ, relative to C/EBPα or C/EBPδ

**Figure 5:**
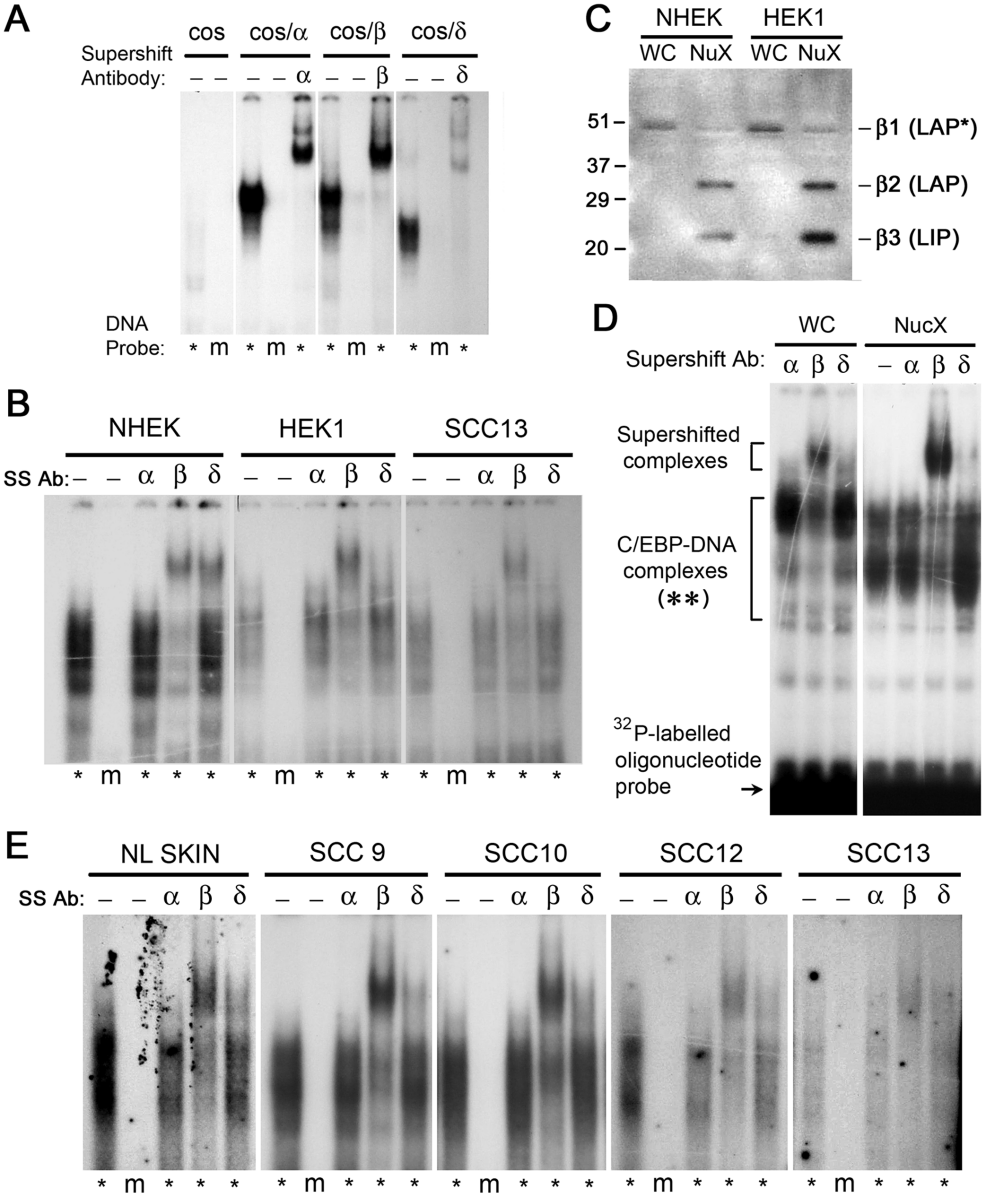
C/EBPβ, and to a lesser extent C/EBPδ, are the predominant C/EBP proteins that bind DNA in human SCC tumors. Protein lysates from cultured cells (A–D) or from tumors (E) analyzed by EMSA (panels A, B, D and E) or by western blot (panel C). Lysates were incubated with a ^32^P-labeled double-stranded DNA probe containing an authentic C/EBP consensus binding sequence (*), or a mutated sequence (m), and the protein-DNA complexes were run on an acrylamide gel and detected by autoradiography. In some lanes, a supershifting antibody to C/EBPα, C/EBPβ, or C/EBPδ, was added to the mixture prior to electrophoresis, as indicated. Lysates for the experiments were: (**A**) Nuclear extracts from cos-7 cells transfected with plasmids encoding full-length C/EBPα (cos/α), C/EBPβ (cos/β), or C/EBPδ (cos/δ); (**B**) Nuclear extracts from normal keratinocytes (NHEK) or from HEK1 or SCC13 cells; (**C**) Nuclear extracts (NucX) or whole-cell (WC) extracts from NHEK or HEK1; this panel is an analysis by western blot for C/EBPβ; (**D**) Nuclear extracts or whole-cell extracts from HEK1, analyzed by EMSA; (**E**) Whole-cell extracts of frozen tissue from SCC specimens previously analyzed in **Fig. 3**. The unbound ^32^P-probe is shown only in panel D.

To determine which C/EBP isoforms in SCC are capable of binding to DNA targets, electrophoretic mobility shift analyses (EMSA) were performed. To validate the techniques, we first analyzed binding of recombinant C/EBPα, C/EBPβ, and C/EBPδ to a DNA oligonucleotide probe harboring a standard C/EBP consensus sequence (**Fig. 5A**). Each of the three C/EBPs bound avidly to DNA, and the protein-DNA complexes were C/EBP site-specific (i.e., failed to bind to a mutant oligonucleotide), and C/EBP protein-specific (i.e., were supershifted by preincubation with anti-C/EBPα, anti-C/EBPβ, or anti-C/EBPδ antisera, respectively). Proteins extracted from the nuclei of SCC cell lines HEK-1 and SCC13 showed mainly C/EBPβ-specific binding, minor binding from C/EBPδ, and no binding from C/EBPα (**Fig. 5B**).

When studying DNA-binding proteins in whole tumors, one must use whole-tissue extracts rather than nuclear extracts because selective extraction of nuclear proteins is impossible (as cells are disrupted during frozen sectioning). To ask whether C/EBPβ expression profiles in whole cell extracts (WC) *vs.* nuclear extracts (NucX) would be similar or different, we compared WC to NucX prepared from normal keratinocytes or from HEK1 carcinoma cells (**Fig. 5C**). NucX contained C/EBPβ-2 and C/EBPβ-3 almost exclusively, whereas WC contained mostly C/EBPβ-1 (**Fig. 5C**). When these two lysates were compared by EMSA (**Fig. 5D**), the abundance of the protein-DNA complexes that formed [see bracket with a double asterisk] tended to reflect the size distribution of C/EBPβ isoforms in the extracts, i.e. larger complexes in WC than in NucX. However, the predominant C/EBP family member expressed was always C/EBPβ, regardless of whether WC or NucX was examined. We next did DNA binding studies on total tissue lysates from normal skin and tumor specimens (**Fig. 5E**). C/EBPβ binding was easily detectable in all specimens. C/EBPδ was only weakly positive, and C/EBPα was absent. In summary, C/EBPβ appears to be the major C/EBP species present and capable of binding conventional C/EBP sites on DNA, both in normal skin and in SSC tumors.

## Discussion

Nonmelanoma skin cancers (NMSC), comprising basal cell carcinomas (BCC) and squamous cell carcinomas (SCC), constitute the most common of all human cancers [51]. SCCs, while representing only 10% of NMSC incidence, are very important because SCC can readily invade and metastasize. Thus an important research goal is to recognize features within tissue biopsies that might identify SCC cancers with the most aggressive biological behavior. In this manuscript, we have examined the expression of three members of the C/EBP transcription factor family that have gained attention as potential prognostic indicators in cancer. Our finding of decreased expression of C/EBPα in SCC concurs with previous reports implicating C/EBPα as a tumor suppressor in myeloid leukemia [8] and cutaneous SCC [14], is consistent with suggestions that low C/EBPα expression in tumors contributes to failure of cell cycle arrest [7], [9], [10], [12], [33]. For C/EBPβ our data agree with some findings, yet contradict other aspects of previous clinicopathological studies on C/EBPβ expression in malignancies of various origins [15], [18], [20], [21]. In agreement, we noted an overall increase in C/EBPβ expression in carcinoma cell lines and in SCC tumors *in vivo* relative to normal keratinocytes. However, these increases were entirely due to elevated amounts of the short isoforms, C/EBPβ−2 and C/EBPβ−3. The full-length isoform C/EBPβ−1 was actually slightly decreased. Because most previous studies were not clear about the exact identity of the large C/EBPβ isoforms being observed, it is not possible to know whether apparent discrepancies reflect differences between skin and other tissues, or instead reflect differences in methodologies for protein separation and detection. In the current study, two additional techniques were employed to validate our histological findings; namely, western blotting was calibrated by the use of recombinant C/EBPβ isoform standards, and EMSA was used to determine functional DNA binding capacity.

Changes in C/EBP expression observed on our western blots cannot be ascribed to differences in cell populations within different tissue specimens, because protein lysates were prepared from frozen biopsy sections with very similar cellular components by histological examination. Thus, in both the normal skin specimens and the SCC tumor sections, >90% of the cells were epithelial (either hair follicles and epidermis, or squamous tumor cells; see (**Fig. S1**). Given these equal proportions of normal and neoplastic epithelial cells, relative changes in C/EBPβ isoforms we observed most likely reflect changes in intracellular C/EBPβ expression, probably at the level of alternative mRNA translation (see Introduction). However, we cannot rule out a role for increased transcription of the C/EBPβ gene, since studies with cultured cells show that ER stress (from glucose deprivation or amino acid deprivation) can induce the expression of human C/EBPβ through unfolded protein response elements in the 3′-UTR of the gene [52], [53]. We also cannot rule out the possibility that post-translational modification of C/EBPβ (via upstream signaling pathways) might affect protein stability and accumulation. Although changes in phosphorylation appeared to be relatively minor, other modifications to these proteins were apparent (see next paragraph).

Changes in differential expression of the C/EBPβ isoforms were among the most interesting findings. C/EBPβ-1 (or LAP*) constituted the vast majority of C/EBPβ expressed in the skin, both normal and SCC, a fact not previously appreciated. Phosphorylation of this isoform at threonine-235 [54] was not significantly altered in SCC specimens (**Fig. 4A**), suggesting that other modifications such as C/EBPβ acetylation, methylation, and sumoylation as reported in other systems [6] might account for shifted C/EBPβ-1 bands that were apparent in SCC (**Fig. 4B**). A recent report by Atwood et al. reported sumoylation of C/EBPβ-1 in breast cancer cells as a possible mechanism to circumvent oncogene induced senescence (OIS) in tumors [55]. Overall, the changes in C/EBPβ-1 were not very impressive, with only a ∼50% decrease observed in the SCC along with modest decreases in post-translational modification (phosphorylation) in well and moderately differentiated SSC. In contrast, C/EBPβ-2 and C/EBPβ-3 (especially C/EBPβ-3) were highly induced in SSC relative to normal skin. Isoform levels correlated directly with levels of PCNA and therefore may reflect cell division within the tumors, either directly or indirectly. Overall, these findings suggest that C/EBPβ-2 and C/EBPβ-3 represent potential biomarkers of proliferative potential in cutaneous SCC.

From the literature, C/EBPβ appears to be involved in tumor cell proliferation through regulation of cyclin D1 and its target genes. Cyclin D1 is often overexpressed in cancers, driving the cell cycle inappropriately and preventing normal G1 arrest. C/EBPβ may be co-regulatory with cyclin D1, both directly and indirectly [29], [56]. In transient transfection studies, C/EBPβ-2 was capable of binding and activating the cyclin D1 gene promoter; which would tend to drive the cell cycle [29]. Another mechanism may involve coregulation of a common set of target genes by C/EBPβ and cyclin D1, as shown by an elegant study in which more than 500 human tumor specimens were examined by gene expression profiling; the C/EBPβ gene was consistently coexpressed with the same set of genes activated by cyclin D1 [56]. The promoters of seven of the cyclin D1-responsive genes that were examined in more detail contained classical C/EBPβ binding sites. However, those promoters were atypical; they were suppressed by wildtype C/EBPβ and activated by a dominant-negative mutant of C/EBPβ (functionally similar to LIP). Thus, C/EBPβ−1 and/or C/EBPβ−2 normally appear to repress cyclin D1 target genes, and cyclin D1 acts by antagonizing this repressor function. In tumors, high levels of dominant-negative C/EBPβ−3 could antagonize the repression of cyclin D1 target genes by displacing the long C/EBPβ isoforms, activating cell cycle progression.

Our *in vivo* data in tumors tend to support previous cell culture and animal experiments demonstrating that C/EBPβ−3 exerts preferential effects upon gene transcription that may favor cancer progression. Zahnow et al. created transgenic mice in which LIP (C/EBPβ-3) was targeted to the mammary gland, leading to hyperplasia and tumorigenesis [57]. Human breast cancer cells lose their ability to undergo growth-arrest in response to TGFβ; Gomis et al. showed that forced overexpresion of LIP exacerbated this loss of TGFβ cytostatic response, whereas C/EBPβ-2 (LAP) overexpression restored the response [17]. Positive correlations between elevated levels of LIP and neoplastic transformation have been reported in murine mammary epithelial tumors [16] and in human breast cancers [29]. Such results argue that a high LIP:LAP ratio is pro-oncogenic whereas a low LIP:LAP ratio favors normal differentiation. The mechanism of action most often quoted involves LIP as a dominant negative inhibitor. Because LIP binds DNA yet lacks a transactivation domain, LIP can displace other activating isoforms (such as LAP) from sites on DNA and block transcription of at least some target genes [26], [57]. However, such effects are gene-dependent since LIP actually activates certain genes in different cellular contexts, as reviewed by Zahnow [6]. Complexities and contradictions abound. For example, C/EBPβ-2 (LAP) when overexpressed at high levels using a retroviral vector, caused neoplastic transformation in human mammary epithelial cells [30], [31], and C/EBPβ-3 (LIP) did not cause transformation and in fact blocked proliferation in that system [31].

C/EBPβ-3 is a relatively minor component when compared to C/EBPβ-1, which presents a puzzle when thinking about how C/EBPβ-3 manages to exert such profound effects during malignant transformation. However, our data suggest that C/EBPβ-3 and C/EBPβ-2 may reside in a different geographic and functional compartment than C/EBPβ-1. In whole-cell lysates of HEK1 cells and NHEK cells, C/EBPβ-1 is the major constituent (**Fig. 5C**). Yet, C/EBPβ-1 is nearly absent in classical nuclear extracts (nuclear proteins extracted using high salt), where C/EBPβ-2 and C/EBPβ-3 are abundant (**Fig. 5C**). Eaton et al. reported substantial C/EBPβ-1 as well as C/EBPβ-2 and C/EBPβ-3 in crude nuclear lysates of normal mammary epithelial cells [29]. In that case, however, the C/EBPβ-1 may have been tightly bound to nuclear/perinuclear membranes or to chromatin, which were spun down in the nuclear pellet prior to collection [29]. The fact that immunostained C/EBPβ is consistently observed in cytoplasmic/perinuclear locations within basal keratinocytes of normal epidermis [47], which contain little or no C/EBPβ-2 nor C/EBPβ-3, suggests that the abundant C/EBPβ-1 isoform resides preferentially in cytoplasmic/perinuclear membranes in those locations. In addition, our western data show that C/EBPβ-2 and C/EBPβ-3 exist in a more loosely bound state than C/EBPβ-1, being preferentially extractable in high salt buffer. C/EBPβ-2 and C/EBPβ-3 may even comprise a majority of the active, DNA-binding C/EBPs detected within SSC tumor lysates, since in the EMSA experiments there is a correlation between (i), expression levels of the individual proteins C/EBPβ-2 and C/EBPβ-3 (**Fig. 3B**) and (ii), intensity of C/EBPβ-containing DNA/protein complexes in the EMSA experiments (**Fig. 5E**); these two parameters are both high for SCC9 and SCC10, and low for SCC12 and SCC13.

The idea that C/EBPβ-1 and C/EBPβ-2 may have different functional roles is consistent with previous studies. C/EBPβ-1 and -2 were each capable of binding to the cyclin D1 promoter, yet only C/EBPβ-2 could activate a cyclin D1 promoter-reporter construct in human mammary epithelial cells [29]. Many other studies showed that C/EBPβ-2 is a stronger transactivator than C/EBPβ-1; reviewed in [6]. Distinct functional and binding properties of C/EBPβ-1 may be attributable to the unique N-terminal region (21 amino acids of C/EBPβ-1), that can specifically bind the SWI-SNF nucleosome remodeling complex [58], and possibly other proteins as well [6].

The potential clinical utility of C/EBPβ-2 and -3 isoforms, as biomarkers for cancer prognosis, will be difficult to evaluate until a more sensitive assay for C/EBPβ isoform detection in routine skin biopsy specimens is developed. Our data, however, suggest that such a developmental effort could be worthwhile. The high correlation between C/EBPβ-3 expression and tumor proliferation in SCC offers promise. On the other hand, very anaplastic tumors may lie so far along a pathway to neoplastic degeneration that normal mechanisms of squamous differentiation no longer apply. Thus, the poorly-differentiated tumors S12 and S13 showed no C/EBPβ-3 elevation, and showed very low proliferation levels.

In summary, we have demonstrated that levels of C/EBPα, C/EBPβ, and to a lesser extent C/EBPδ are decreased in human SSC. Observed losses of C/EBPα are in accord with the widely acknowledged tumor suppressor function of C/EBPα, now well-established in myeloid leukemia and in some solid tumors including SCCs [4], [7], [59]. More novel is our finding that C/EBPβ-1 (the most abundant C/EBPβ isoform) is also reduced in skin carcinomas, a fact not previously recognized. Most interesting, however, is our demonstration of a robust and qualitative induction of C/EBPβ-3 which correlates with proliferative activity and could contribute to gene dysregulation in SSC tumors. Our analysis of DNA-binding activity suggests that C/EBPβ isoforms constitute most of the functional C/EBP family proteins in SCC. Combined with experimental evidence from other systems, these data further strengthen the possibility that C/EBPβ-3 (and maybe also C/EBPβ-2) are important players in aberrant gene regulation in carcinomas, and should be investigated as potentially useful markers of neoplastic progression in SCC. In other words, the short C/EBPβ proteins should be on the short list of future biomarker studies.

## Supporting Information

Figure S1 **Histological grading of the human SCC tumors analyzed by Western blot and EMSA in** **Figs 3** **–** **5** **.** Post-fixed frozen skin sections from samples of normal skin, and five squamous cell carcinomas (SCC9 to SCC13), were stained with hematoxylin and eosin (H&E). These were the specimens amongst the specimens in Table 1 that were large enough to provide sufficient material for Western blot and DNA-binding analyses. The fields shown here illustrate the following: in normal skin, the appearance of normal hair follicles (*hf*) and normal keratinocytes within a follicular root sheath (*arrowheads*); in squamous tumors SCC9 to SCC11, the presence of cells with an epithelial morphology and eosinophilic keratinizing islands (*ker*); in SCC12, unusual clear-cell changes; and in SCC13, nuclear pleomorphism and a marked stromal response. *Scale bar*, 100 µm. A histological grade for each SCC specimen, S9 to S13, was determined by two board-certified dermatopathologists (Drs. MP and SB). Tumors were scored as well-differentiated (*well*), moderately-differentiated (*mod*), or poorly-differentiated (*poor*) using histopathologic criteria such as the presence/absence of keratinization, nuclear pleomorphism, stromal reaction, etc. The relative concordance of the two pathologists’ scores is indicated on each image. S9 was an extensive tumor on the back, untreated for years. S13 was a metastatic tumor on the face.
(TIF)

Figure S2 **Semiquantitative analysis of relative differences in immunofluorescence, using micrographs of normal skin and tumor tissue, captured as a continuous series of timed exposures.** Imaging of fluorescence intensity was standardized as follows. Seven different exposures of a given tumor specimen, and a normal skin specimen from the same patient were digitally captured on an Olympus BX50 fluorescent microscope (Olympus America, Center Valley, PA) equipped with a CoolSNAP-Pro color CCD camera (Media Cybernetics, Bethesda, MD). Each exposure time in the series doubled the amount of collected light. Pairs of digital images from tumor and normal hair follicles were compared side-by-side on a computer monitor, and the two images with the most closely matched intensities were noted. The relative difference in C/EBP protein between tumor and normal was determined from these exposure times. This analysis assumes that fluorescent signal is directly proportional to C/EBP protein bound by primary antibody, since no amplification step was employed during immunostaining. Two examples of this analytic approach are shown below. For C/EBPα: The best match (equal intensities) is for the normal follicle at 2.0 s and tumor at 6.4 s. From the ratio of exposure times (2.0/6.4), the tumor is ∼0.3 the intensity of normal tissue, or ∼3-fold less intense than the normal hair follicle. For C/EBPβ: The best match is the normal follicle at 2.0 s and the tumor at 3.2 s. (Higher exposure times were in the saturation range, and therefore less reliable). From the ratio of exposure times (2.0/3.2), the tumor intensity is ∼0.63 or 63% of the normal tissue intensity, a reduction in intensity of approximately one-third. (Scale bar: 100 µm.)
(TIF)

Figure S3 **Immunohistological analyses of differentiation and proliferation in normal skin and SCC tumors.** Methanol fixed frozen sections from normal skin and SCC tumors (two tumors each) were stained with E-cadherin (E-cad) and Ki67, markers of differentiation and proliferation, respectively. **(A)** Membranous expression of E-cad in cells in epidermis (normal skin; left panel), in tumor nests from well to moderate differentiated (middle panel) and poorly differentiated SCCs (right panel). Note the increase in E-cad expression associated with well to moderate differentiated state of the SCC. **(B)** Nuclear expression of Ki67 in cells of basal layer in epidermis (normal skin; left panel), in tumor nests from well to moderate differentiated (middle panel) and poorly differentiated SCCs (right panel). Note the increase in Ki67 expressing cells in SCCs, a marker routinely used by dermatopathologists. Scale bar, 50 µM.
(TIF)
