# Supplementary figures and images for: C/EBP Transcription Factors in Human Squamous Cell Carcinoma: Selective Changes in Expression of Isoforms Correlate with the Neoplastic State
Source: PLoS One. 2014 Nov 17;9(11):e112073. doi: 10.1371/journal.pone.0112073 (PMC4234316; doi:10.1371/journal.pone.0112073)

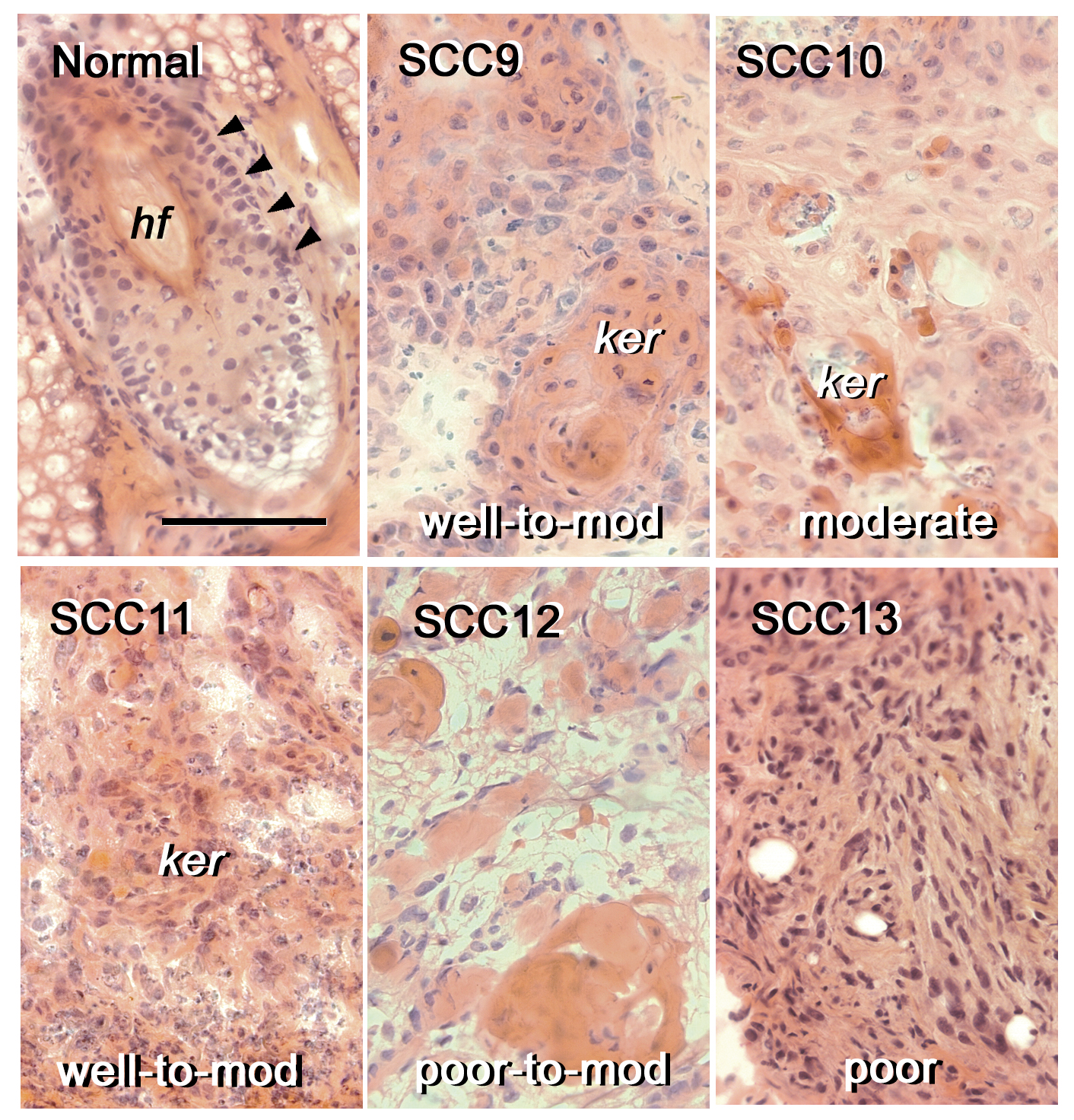

Supplement: Figure S1 — Histological grading of the human SCC tumors analyzed by Western blot and EMSA in Figs 3 – 5 . Post-fixed frozen skin sections from samples of normal skin, and five squamous cell carcinomas (SCC9 to SCC13), were stained with hematoxylin and eosin (H&E). These were the specimens amongst the specimens in Table 1 that were large enough to provide sufficient material for Western blot and DNA-binding analyses. The fields shown here illustrate the following: in normal skin, the appearance of normal hair follicles (hf) and normal keratinocytes within a follicular root sheath (arrowheads); in squamous tumors SCC9 to SCC11, the presence of cells with an epithelial morphology and eosinophilic keratinizing islands (ker); in SCC12, unusual clear-cell changes; and in SCC13, nuclear pleomorphism and a marked stromal response. Scale bar, 100 µm. A histological grade for each SCC specimen, S9 to S13, was determined by two board-certified dermatopathologists (Drs. MP and SB). Tumors were scored as well-differentiated (well), moderately-differentiated (mod), or poorly-differentiated (poor) using histopathologic criteria such as the presence/absence of keratinization, nuclear pleomorphism, stromal reaction, etc. The relative concordance of the two pathologists’ scores is indicated on each image. S9 was an extensive tumor on the back, untreated for years. S13 was a metastatic tumor on the face. (TIF) [file pone.0112073.s001.tif]

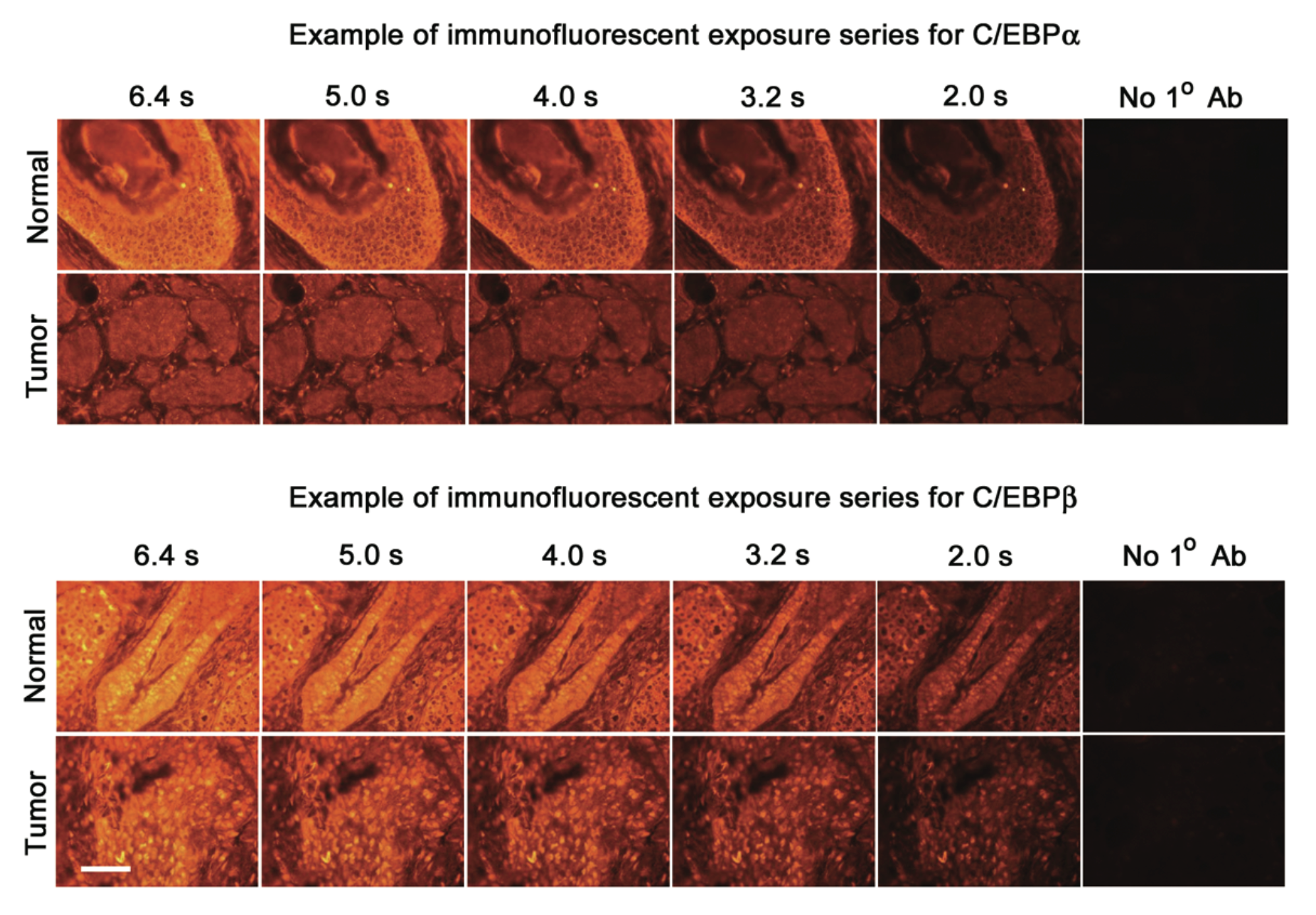

Supplement: Figure S2 — Semiquantitative analysis of relative differences in immunofluorescence, using micrographs of normal skin and tumor tissue, captured as a continuous series of timed exposures. Imaging of fluorescence intensity was standardized as follows. Seven different exposures of a given tumor specimen, and a normal skin specimen from the same patient were digitally captured on an Olympus BX50 fluorescent microscope (Olympus America, Center Valley, PA) equipped with a CoolSNAP-Pro color CCD camera (Media Cybernetics, Bethesda, MD). Each exposure time in the series doubled the amount of collected light. Pairs of digital images from tumor and normal hair follicles were compared side-by-side on a computer monitor, and the two images with the most closely matched intensities were noted. The relative difference in C/EBP protein between tumor and normal was determined from these exposure times. This analysis assumes that fluorescent signal is directly proportional to C/EBP protein bound by primary antibody, since no amplification step was employed during immunostaining. Two examples of this analytic approach are shown below. For C/EBPα: The best match (equal intensities) is for the normal follicle at 2.0 s and tumor at 6.4 s. From the ratio of exposure times (2.0/6.4), the tumor is ∼0.3 the intensity of normal tissue, or ∼3-fold less intense than the normal hair follicle. For C/EBPβ: The best match is the normal follicle at 2.0 s and the tumor at 3.2 s. (Higher exposure times were in the saturation range, and therefore less reliable). From the ratio of exposure times (2.0/3.2), the tumor intensity is ∼0.63 or 63% of the normal tissue intensity, a reduction in intensity of approximately one-third. (Scale bar: 100 µm.) (TIF) [file pone.0112073.s002.tif]

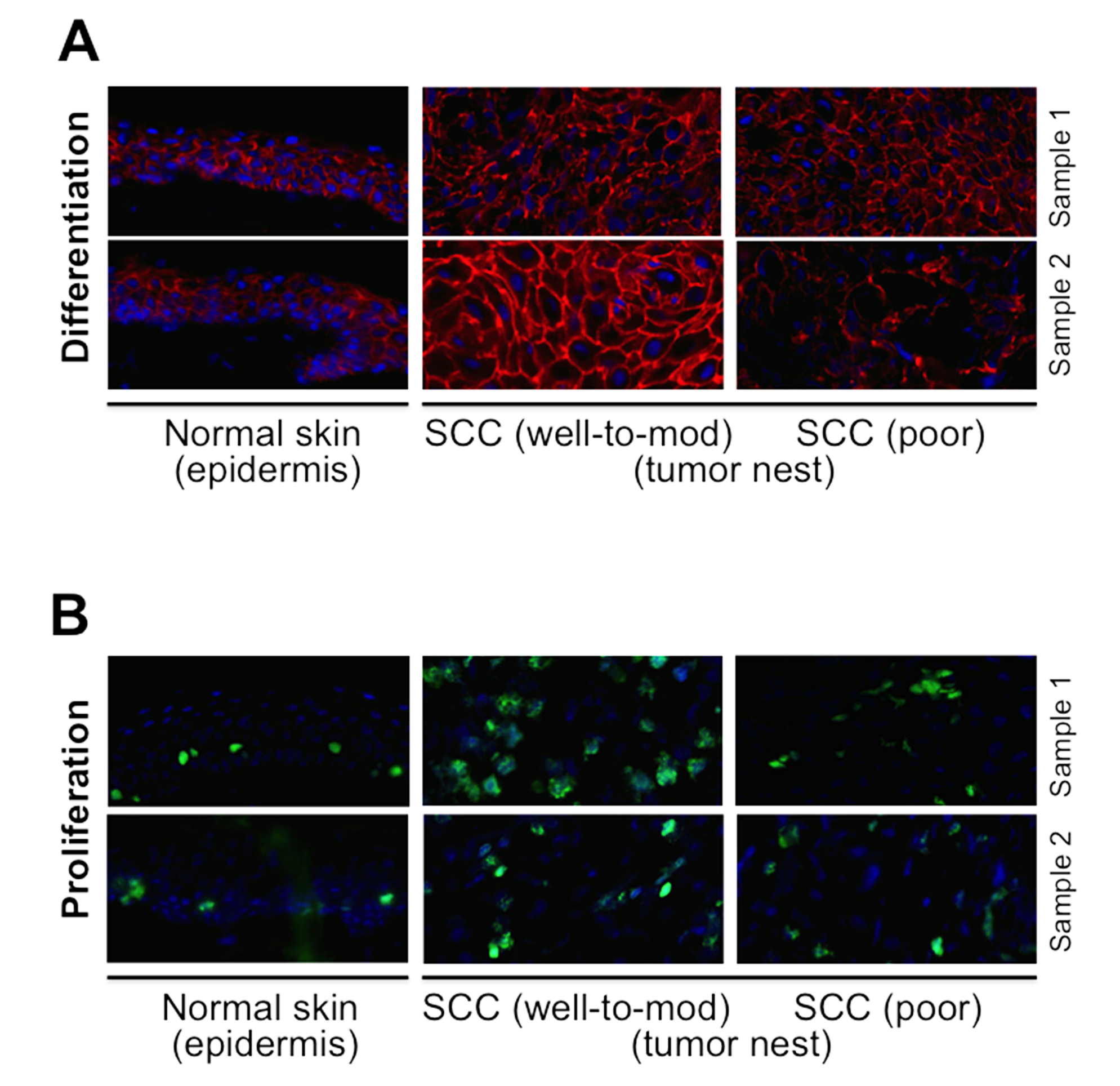

Supplement: Figure S3 — Immunohistological analyses of differentiation and proliferation in normal skin and SCC tumors. Methanol fixed frozen sections from normal skin and SCC tumors (two tumors each) were stained with E-cadherin (E-cad) and Ki67, markers of differentiation and proliferation, respectively. (A) Membranous expression of E-cad in cells in epidermis (normal skin; left panel), in tumor nests from well to moderate differentiated (middle panel) and poorly differentiated SCCs (right panel). Note the increase in E-cad expression associated with well to moderate differentiated state of the SCC. (B) Nuclear expression of Ki67 in cells of basal layer in epidermis (normal skin; left panel), in tumor nests from well to moderate differentiated (middle panel) and poorly differentiated SCCs (right panel). Note the increase in Ki67 expressing cells in SCCs, a marker routinely used by dermatopathologists. Scale bar, 50 µM. (TIF) [file pone.0112073.s003.tif]
